# Response to: the nature of evidence for and against epigenetic inheritance

**DOI:** 10.1186/s13059-015-0714-1

**Published:** 2015-07-11

**Authors:** Piroska E. Szabó

**Affiliations:** Van Andel Research Institute, Center for Epigenetics, 333 Bostwick Ave, Grand Rapids, MI 49503 USA

## Abstract

We thank Dr. Nadeau for his interest in our work. Dr. Nadeau has raised concerns about the experimental approach (mouse strains, route of administration, lack of phenotypic assessment) and about the validity of our conclusions. We will respond to each of these concerns point-by point.

Please see related article: www.dx.doi.org/10.1186/s13059-015-0709-y

## Genetic background

We used inbred 129 or FVB female G0 mice for treatment. In our opinion, inbred strains should be preferred for identifying epigenetic mechanisms without confounding effects of genetic diversity. Indeed, the inbred 129 mouse strain was shown previously to exhibit transgenerational (G1-G3) apoptotic spermatogenic cell defect phenotype after vinclozolin exposure, as well as outbread CD-1 mice [1]. In another study, inbred FVB mice were used to show that vinclozolin administration induced trangenerational alterations in both maternally and paternally imprinted genes in offspring [2, 3].

## Treatment protocols

In our opinion, oral treatment better reflects human exposure to endocrine disrupters (EDs). Additionally, gavage gives the best control over exposure dose and also gives more uniform ED distribution between fetuses than intraperitoneal injection to pregnant dams. Because we measured genome-wide transcription changes in G1 prospermatogonia after oral exposure to G0 dams, we feel confident that the EDs have reached the G1 prospermatogonia using our treatment protocols, allowing us to test whether any aberration persisted into the G2 germ cells.

## Lack of phenotypic assessment

Our goal was not to reproduce or disprove gross phenotypic changes described by others. We wanted to detect direct changes in fetal germ cells at the level of transcription or DNA methylation, and to test whether such aberrations persisted into the germ cells of the next generation. Other mechanisms such as histone modifications, histone variants, and noncoding RNAs also participate in gene regulation and may transmit epigenetic aberrations between generations. However, it is reasonable to expect that such aberrations would also manifest in altered gene expression patterns. Therefore, we considered transcription changes as phenotypic readouts of putative epigenetic changes. Similarly, transgene expression (at the protein and mRNA levels) served as phenotypic readout in the paper cited by Dr. Nadeau [4]. Importantly, the transcription changes that we detected were very specific and reflected the nature of the endocrine disruptors, but these did not persist into the next generation. Indeed, in the small number of cases when transcription change was detected both in G1 and G2, it was overcompensated in G2: a significantly greater number of the common changes occurred in the opposite direction.

The aim of our study was to systematically and rigorously evaluate the effects of EDs on global epigenetic reprogramming in the male mouse germ line after *in utero* exposure. We wanted to tackle the bottleneck between generations: everything --genetic or epigenetic-- that is inherited from one generation to the next must go through the gamete. To detect the immediate epigenetic damage caused by *in utero* ED treatment, one has to search in the germ cells at the time of exposure. To detect persistent changes between generations, one has to examine the exposed germline and the germline of the next generation. Previous efforts toward understanding the mechanism underlying transgenerational inheritance (TGI) after *in utero* vinclozolin treatment found DNA methylation changes in the adult testis in G1, G2, and G3 with partial penetrance [5], but this experiment did not investigate purified germ cells. In two other experiments, G3 primordial germ cells and G3R prospermatogonia from rat embryos/fetuses and G3R adult sperm were analyzed using MeDIP and/or Affymetrix microarrays to identify persistent vinclozolin-dependent inheritance [6, 7], but these studies did not test for immediate changes in G1R prospermatogonia. In the current study, we were unable to reproduce the reported methylation changes using the MIRA-chip: the top hits of that paper did not, in our data set, display methylation changes or lacked DNA methylation completely. Other studies have reported aberrant transcription and DNA methylation in the soma of the G3 unexposed generation, claiming evidence for TGI without assessing the aberrations in the G1 germline [8, 9]. There was no overlapping hit between the 11 organs, suggesting to us that those hits likely did not originate from the germline.

Among epigenetic mechanisms, we searched for immediate and persistent DNA methylation changes. Regulatory RNAs and histone composition provide additional layers to gene regulation. In a very recent study, the Lin28/let-7/BLIMP1 pathway was shown deregulated in F1-F2-F3 13.5 dpc male mouse germ cells after vinclozolin exposure in utero [10]. However, the phenotypes that can be expected to derive from such immediate and persistent epigenetic aberrations (reduced number of primordial germ cells and germ cell apoptosis at 13.5 dpc) did not prevail beyond F2.

In another example, nuclear small RNA/chromatin pathway can maintain transgene repression for many generations in the germline of *C. elegans* [4]. However, this mechanism involves secondary siRNA, and evidence for secondary RNA pathways appear to be missing in mammals. This mechanism cannot explain the adult onset of somatic TGI phenotypes [9], because the secondary siRNA-mediated repression cannot exit the germline. *C. elegans* provides a unique experimental system for studying TGI, but one has to keep in mind the fundamental differences that exist between the *C. elegans* and mammalian systems. The short lifespan of *C. elegans* allows the survival of specific molecules from one generation to the next, the use of hermaphrodites circumvents dilution problems that come with sexual reproduction, and unlike in mammals, the germ line is a separate lineage starting at the first cell division.

As Dr. Nadeau suggests, one should also consider the possibility of a step-wise mechanism consisting of initiation and propagation. However, evidence for such events mediating TGI phenotype in mammals is still missing. Altered miRNA expression was detected in the early life trauma-treated mouse sperm (referred to as F1) but not in F2 [11]. According to the proposed model, the phenotype in F3 must be relayed by an additional epigenetic mechanism, for example DNA methylation. The mechanistic link, however, still has to be proven between F1 miRNA and F2 DNA methylation, or between any of these epigenetic marks and the somatic phenotype. In the case when F1-F4 phenotypes are identical [5], one would expect the underlying molecular mechanisms to be also very similar.

We would like to maintain our conclusions. We feel that our conclusions reflect exactly what we have found. Our conclusions were specific to transgenerational epigenetic inheritance (TGI) of ED-triggered epigenetic changes in mammals, and suggest that ED exert direct epigenetic effects in exposed fetal germ cells, which are corrected by reprogramming events in the subsequent generation.

## Abbreviations

ED: Endocrine disruptor
miRNA: MicroRNA
siRNA: Small interfering RNA
TGI: Transgenerational epigenetic inheritance
